# Wine glass size and wine sales: a replication study in two bars

**DOI:** 10.1186/s13104-017-2610-0

**Published:** 2017-08-01

**Authors:** Rachel Pechey, Dominique-Laurent Couturier, Gareth J. Hollands, Eleni Mantzari, Zorana Zupan, Theresa M. Marteau

**Affiliations:** Behaviour and Health Research Unit, Institute of Public Health, University of Cambridge, Cambridge, UK

**Keywords:** Wine, Glass size, Purchasing, Portion size, Alcohol, Replication, Multiple treatment reversal design

## Abstract

**Objective:**

Wine glass size may influence perceived volume and subsequently purchasing and consumption. Using a larger glass to serve the same portions of wine was found to increase wine sales by 9.4% (95% CI 1.9, 17.5) in a recent study conducted in one bar. The current study aimed to replicate this previous work in two other bars using a wider range of glass sizes. To match the previous study, a repeated multiple treatment reversal design, during which wine was served in glasses of the same design but different sizes, was used. The study was conducted in two bars in Cambridge, England, using glass sizes of 300, 370, 510 ml (Bar 1) and 300 and 510 ml (Bar 2). Customers purchased their choice of a 750 ml bottle, or standard UK measures of 125, 175 or 250 ml of wine, each of which was served with the same glass.

**Results:**

*Bar 1* Daily wine volume (ml) purchased was 10.5% (95% CI 1.0, 20.9) higher when sold in 510 ml compared to 370 ml glasses; but sales were not significantly higher with 370 ml versus 300 ml glasses (6.5%, 95% CI −5.2, 19.6). *Bar 2* Findings were inconclusive as to whether daily wine purchased differed when using 510 ml versus 300 ml glasses (−1.1%, 95% CI −12.6, 11.9). These results provide a partial replication of previous work showing that introducing larger glasses (without manipulating portion size) increases purchasing. Understanding the mechanisms by which wine glass size influences consumption may elucidate when the effect can be expected and when not.

*Trial registration* This study is a replication study, based on the procedure set out in the trial registration for the study that it attempts to replicate (ISRCTN registry: ISRCTN12018175)

## Introduction

Excessive alcohol consumption is a major contributor to the global burden of disease [1]. Results of a Cochrane review showed tableware size influences consumption for food and non-alcoholic beverages, but there was an absence of evidence relating to alcoholic beverages [2]. This paper explores glassware as a potential cue that influences alcohol consumption.

Several studies indicate that larger glasses may lead to more alcohol being poured (including pouring by serving staff such as bartenders), and subsequently consumed [3, 4]. In addition, the same portion of wine served in a larger glass may be perceived as less than when served in a smaller glass [5]. Together, these studies suggest that serving alcohol in larger glasses might increase consumption.

The effects of wine glass size on purchasing (a proxy measure of consumption) was examined in one recent study conducted in a bar setting [6]. This study, carried out in one establishment in Cambridge, England, suggested that using larger (370 ml) glasses increased wine sales by 9.4% compared to serving wine in the bar’s standard glasses (300 ml). However, results were inconclusive comparing sales using smaller glasses (250 ml) versus standard glasses. The current study aims to replicate this previous study in two further bars.

## Main text

### Study design

In order to try to replicate the previous study examining the impact of glass size on purchasing [6], glass size was changed over fortnightly periods in each of two bars using a multiple treatment reversal design (see Table 1).

**Table 1:** Glass size manipulations

	Bar 1 (ml)	Bar 2
Fortnight 1	370	300 ml
Fortnight 2	510	510 ml^a^
Fortnight 3	370	300 ml
Fortnight 4	300	510 ml
Fortnight 5	370	300 ml
Fortnight 6	510	510 ml^b^
Fortnight 7	370	300 ml
Fortnight 8	300	–
Fortnight 9	370^c^	–

^a^During the week following this period, the venue was closed for refurbishment for 4 days: data from this week were not included in the study

^b^This period was continued for an additional week as protocol violations (mixed glass sizes used due to large numbers of customers) were identified in the 1st week, which consequently was not included in the analysis

^c^The 3 last days of this fortnight were not included in the analysis due to protocol violations (mixed glass sizes used due to large numbers of customers, as a result of a festival occurring close to the venue)

The smallest glass used in the previous study (250 ml) could not be used in this study, as the glasses needed to be able to hold 250 ml portions (sold in these bars). A larger glass (510 ml) was included instead. In Bar 1, in addition to the 510 ml glass, 300 and 370 ml glasses were used, as in Pechey et al. [6]. In Bar 2, only the largest and smallest glasses (300 and 510 ml) were used, due to limited time during which the study could be run in this bar.

The primary outcome was the daily volume of wine (ml) purchased, with reference groups being 370 ml for Bar 1 and 300 ml for Bar 2.

### Intervention

Glasses were changed over fortnightly periods to alternates of the same design but which varied in size. In keeping with UK legal requirements [7] both venues served wine either by the bottle (750 ml), or by the glass in three specified quantities (125, 175, 250 ml). Both bars used one glass size for all portions (pre-intervention and during the intervention).

The glasses used in the study matched those used in Pechey et al. [6], namely unlined Royal Leerdam glasses of varying capacity.

Pre-intervention, the glasses used in the two bars were: *Bar 1* Reserva, triple-lined, 350 ml; *Bar 2* Cabernet Tulip, triple-lined, 350 ml.

### Setting

The study was conducted in two bars in Cambridge, England, between March and July 2016. Table 2 shows the characteristics of the bars and interventions both in the current study and the original study [6].

**Table 2:** Characteristics of study bars and interventions

	Bars		
	Pechey et al. [6] Bar	Bar 1	Bar 2
Study Bars [mean (sd)]			
Price of 175 ml of wine [£ ($/€)]	5.00 (~6.2 USD/5.9 EUR)	4.10 (~5.1 USD/4.8 EUR)	5.40 (~6.7 USD/6.4 EUR)
Wine sales (litres/week)	121.0 (12.6)	91.5 (15.2)	100.7 (14.1)
Wine sales as proportion of total sales (%)	9.9	7.8	7.3
Wine sales by bottle (%)	22	12	15
Wine sales by glass by portion (%)			
125 ml	10	35	0^a^
175 ml	90	27	52
250 ml	–	38	48
Mean portion sold by glass (ml)	170	186	211
Intervention			
Standard glass size (ml)	300	350	350
Intervention glass sizes (ml)	250, 300, 370	300, 370, 510	300, 510
Study period	March–July 2015	March–July 2016	March–July 2016

^a^In Bar 2, 125 ml portions were only available on request, and none were sold during the study period

### Procedure

Glasses were changed by bar staff in each of the two bars on Monday mornings each fortnight throughout the study period. Email reminders were sent by a researcher at 8 a.m. on the morning when a change of glasses was needed, stating the size of glass to be used for the ensuing fortnight. Fidelity to protocol was checked by a researcher visiting the bars at the start and end of each intervention period. No changes were made to the wine menus or pricing during the study period. Sales data were obtained from the till records of the two bars.

### Analysis

Separate regression analyses for each venue were used to predict the log of the daily wine sales volume (in ml) from glass size. Analyses controlled for the busyness of the venue, as measured by the log of their daily sales of products excluding wine. Dummy variables indicating day of the week and month controlled for weekly and seasonal time trends. Weather variables (daily temperature at 5 p.m., daily rainfall, daily minutes of sunshine) were also considered. Finally, given the impact of major sports events on alcohol sales, the period during which the 2016 UEFA European Championship was underway was also controlled for. Data from periods during which protocol violations were identified were excluded from the analysis. As in Pechey et al. [6], both the mean and variance of wine sales volume were modelled, due to heteroscedasticity.

## Results

Table 3 shows the unadjusted mean sales volume for each bar under the different glass size conditions, with a linear pattern of increasing sales with increasing glass size seen in Bar 1, and the opposite suggested in Bar 2.

**Table 3:** Daily wine sales (litres) for each bar, by glass size [mean (sd)]

	Bar 1	Bar 2
300 ml glass	12.4 (7.3)	14.9 (9.7)
370 ml glass	12.7 (6.7)	–
510 ml glass	14.0 (8.0)	13.7 (8.8)

Protocol violations were identified on two occasions (once in each bar), where bars used different glass sizes simultaneously due to larger than usual numbers of customers. As a result, these periods were excluded from analyses.

**Fig. 1:**
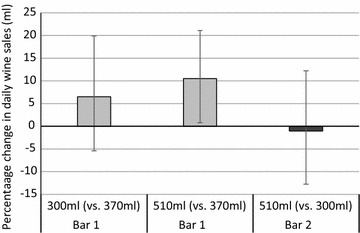
Daily wine sales (ml) for each glass comparison. Error bars show 95% CIs; Reference glass in each comparison indicated in *parentheses*

Figure 1 presents the results of the main analyses for Bars 1 and 2, controlling for the aforementioned possible confounders. In Bar 1, daily wine sales were not significantly higher when using the 370 ml compared to 300 ml glasses (6.5% sales increase, 95% CI 5.2% decrease, 19.6% increase). However, wine sales in this venue were 10.5% (95% CI 1.0%, 20.9%) higher when using 510 ml glasses compared to 370 ml glasses. For Bar 2, findings were inconclusive for the comparison between 510 ml and 300 ml glasses (1.1% sales decrease with 510 ml glasses, 95% CI 12.6% decrease, 11.9% increase).

## Discussion

In Bar 1, sales were approximately 10% higher when wine was served using 510 ml glasses, compared to the 370 ml glasses, a similar increase in sales as in Pechey et al. [6]. However, there was no statistically significant difference in sales between periods using the 370 and 300 ml glasses in Bar 1, in contrast to the findings of the previous study, where the significant sales increase was observed when using 370 ml glasses compared to 300 ml glasses [6]. Moreover, in Bar 2 no significant differences were observed in sales when using the 510 ml compared to the 300 ml glasses. Overall, the results of the current study provide only a partial replication of previous findings.

There are several possible explanations for why serving wine in larger wine glasses is sometimes associated with increased consumption and sometimes not. First, there may be no true effect and what we are observing is random fluctuation. While possible, this may be less likely given that each significant result is in the same direction—i.e. indicating that larger glasses increase purchasing or consumption—in both the current study and in Pechey et al. [6]. Moreover, this directionality complements previous evidence from the Cochrane review showing tableware size increases consumption for non-alcoholic beverages [2].

Second, it may be that what we are observing is not a main effect but rather an interaction between the wine glass size and the portion size it contains. One key difference between the studied bars is the typical portion sizes served: while in Pechey et al. [6] the mean portion size was 170 ml, for Bar 1 of the present study the mean portion size sold was 186 ml and for Bar 2, it was 211 ml. Previous work looking at perceptual differences when presenting 125, 175 and 250 ml portions in smaller and larger glasses suggested that as portion sizes increase, perceived differences between portions by glass size decrease [5]. Thus, the perceptual difference between different sized glasses (e.g. 300 ml vs. 370 ml) containing larger portions (250 ml) may be smaller than the perceptual difference between smaller portions (175 ml) in the same glasses (7). As such, any effect that we observed when comparing glass sizes of 300 and 370 ml in a bar where the most frequently served portion size was 175 ml (i.e. in Pechey et al. [6]) may not be apparent if we instead looked at these glasses in a bar where the most frequently served portion size is 250 ml (as in Bar 1 in the current study). If portion size does alter any effects of glass size, this could potentially influence all of the glass comparisons investigated in both the current study and in Pechey et al. [6], and could explain why effects of glass size are only observed for certain comparisons. However, any relationship between glass size and portion size is unlikely to be linear (given portions greater than 0.5 have been shown to be underestimated, with the degree of underestimation increasing as proportions approach 1 [8, 9]). As such, there remains considerable uncertainty about how portion size might interact with glass size, and thus whether these effects would account for the pattern of results observed in this study.

### Limitations

Given the paucity of evidence regarding the impact of glass size on wine purchasing and consumption, this study aimed to replicate the only study to address this to date. By using the same glass design in the same English city during the same time of the year, this study provides a strong initial assessment of the reliability of the effect of wine glass size on purchasing. In addition, the current study goes beyond the original by examining a greater range of glass size comparisons.

There are, however, several limitations to the current study. The study examined purchasing for on-site consumption rather than consumption *per se*. In addition, to replicate the original study, this study focused on bars in the same city, which limits the generalisability of any results beyond one relatively affluent area of England. Other characteristics varied between bars, and may limit comparability (e.g. average price of wine). Finally, as the study focused on sales at the level of the establishment, characteristics of the patrons at the different sites were not examined. Exploring sales at the individual level could also have established the average length of customers’ visits: shorter visits may make it harder to observe any impact of glass size in bars’ sales data (although it is possible that there was an effect on consumption for patrons over that evening).

### Implications for research and policy

Further research is needed to establish the validity of the suggested explanation of the results. Firstly, examining perceptual differences by portion size for the glass size comparisons used in this study would provide further evidence as to the nature of any interaction between these variables. Secondly, examining the purchasing and consumption behaviours of individuals over time in a bar setting would allow explorations of mechanisms underlying any increases in consumption (e.g. speed of consumption), as well as possible limitations to these effects (e.g. larger portion sizes). This would add to the small but growing literature looking at micro-drinking behaviours relating to shape and size of glasses on consumption of alcohol [10, 11].

While further research needs to establish the nature of any limitations to the effects of wine glass size upon consumption, if this does prove to be a reliable effect under certain conditions, then possible means of implementing interventions targeting glass size could be considered as part of existing effective and cost-effective alcohol control policies [12], including local licensing.

## Conclusions

These results provide a partial replication of the original study showing using larger glasses (without manipulating portion size) increases purchasing [6]. The pattern of results observed across both the original and current study may reflect the effects of an interaction between wine glass size and wine portion size upon purchasing and consumption. While further work is necessary to test this hypothesis, these results suggest reductions in glass sizes could reduce consumption, albeit effective only under certain conditions.
