# Erratum to: Wine glass size and wine sales: a replication study in two bars

**DOI:** 10.1186/s13104-017-2759-6

**Published:** 2017-08-29

**Authors:** Rachel Pechey, Dominique-Laurent Couturier, Gareth J. Hollands, Eleni Mantzari, Zorana Zupan, Theresa M. Marteau

**Affiliations:** Behaviour and Health Research Unit, Institute of Public Health, University of Cambridge, Cambridge, UK

## Erratum to: BMC Res Notes (2017) 10:287 DOI 10.1186/s13104-017-2610-0

Following publication of the original article [1], the authors requested the following changes:

- In the abstract, results: Previous phrasing “Bar 1: Daily wine volume (ml) purchased was 10.5% (95% CI 1.0, 20.9) higher when sold in 510 ml compared to 370 ml glasses; but sales were not significantly higher with 370 ml vs. 300 ml glasses (6.5%, 95% CI −5.2, 19.6)” to become “Bar 1: Daily wine volume (ml) purchased was 10.5% (95% CI 1.0, 20.9) higher when sold in 510 ml compared to 370 ml glasses; but sales were not significantly different with 300 ml vs. 370 ml glasses (6.5%, 95% CI −5.2, 19.6)”.
- In the results section, paragraph 3: Previous phrasing “In Bar 1, daily wine sales were not significantly higher when using the 370 ml compared to 300 ml glasses (6.5% sales increase, 95% CI 5.2% decrease, 19.6% increase)” to become “In Bar 1, daily wine sales were not significantly different when using the 300 ml compared to 370 ml glasses (6.5% sales increase, 95% CI 5.2% decrease, 19.6% increase)”.

The original article has been corrected.
